# A critical assessment of sparse PCA (research): why (one should acknowledge that) weights are not loadings

**DOI:** 10.3758/s13428-023-02099-0

**Published:** 2023-08-01

**Authors:** S. Park, E. Ceulemans, K. Van Deun

**Affiliations:** 1 Tilburg University, Methods and Statistics, Tilburg, The Netherlands; 2 KU Leuven, Psychology and Educational Sciences, Leuven, Belgium

## Abstract

Principal component analysis (PCA) is an important tool for analyzing large collections of variables. It functions both as a pre-processing tool to summarize many variables into components and as a method to reveal structure in data. Different coefficients play a central role in these two uses. One focuses on the *weights* when the goal is summarization, while one inspects the *loadings* if the goal is to reveal structure. It is well known that the solutions to the two approaches can be found by singular value decomposition; weights, loadings, and right singular vectors are mathematically equivalent. What is often overlooked, is that they are no longer equivalent in the setting of sparse PCA methods which induce zeros either in the weights or the loadings. The lack of awareness for this difference has led to questionable research practices in sparse PCA. First, in simulation studies data is generated mostly based only on structures with sparse singular vectors or sparse loadings, neglecting the structure with sparse weights. Second, reported results represent local optima as the iterative routines are often initiated with the right singular vectors. In this paper we critically re-assess sparse PCA methods by also including data generating schemes characterized by sparse weights and different initialization strategies. The results show that relying on commonly used data generating models can lead to over-optimistic conclusions. They also highlight the impact of choice between sparse weights versus sparse loadings methods and the initialization strategies. The practical consequences of this choice are illustrated with empirical datasets.

## Introduction

“Principal component analysis (PCA) is probably the most popular multivariate statistical technique and it is used by almost all scientific disciplines. It is also likely to be the oldest multivariate technique.” (Abdi & Williams, 2010, p.433). Often referred to as the basis for multivariate data analysis (Wold et al., 1987), the central idea of PCA is to reduce a possibly large set of variables to a few derived variables - usually called components - which preserve a maximum amount of information in the data (Jolliffe, 2002). The resulting low-dimensional representations are mainly used in two ways: they are either used as a data *pre-processing step* where the constructed summary scores are subsequently adopted for regression or classification or as an *exploratory tool* to detect patterns and to create attractive visualizations of the data (Gabriel, 1971). PCA produces two types of coefficients that serve these aims: variable ‘weights’ that define the transformation from the raw data to the summary scores and ‘loadings’ which reflect the strength of association of the raw variables with the low-dimensional representations. Although PCA has been presented in several ways, it is well known that the different PCA solutions are equivalent and that weights and loadings can both be obtained from the singular value decomposition (SVD) of the data matrix.

With the advent of big data, especially those in which the number of variables largely exceeds the number of observation units, the use of PCA to reduce the dimensionality of the data has become more widespread. However, there are several issues with using PCA in the high-dimensional setting. First, computation of weights and loadings may suffer from a problem of statistical inconsistency in high-dimensional data settings (Johnstone & Lu, 2009; Shen et al., 2016). Furthermore, interpreting the summarized scores via inspection of weights and loadings becomes difficult as PCA computes these coefficients for the entire set of variables. Traditionally, the burden of interpretation that arises from studying all of the coefficients had been addressed by rotation to simple structure Jolliffe (2002). Yet, rotation followed by neglecting coefficients with small magnitude has been pointed out to be a rather arbitrary and suboptimal way of selecting variables (Cadima & Jolliffe, 1995; Trendafilov & Adachi, 2015).

In response to these issues, sparse versions of PCA that reduce the number of variables involved in the PCA representation have been proposed (Jolliffe et al., 2003; Zou et al., 2006; Shen & Huang, 2008; Witten et al., 2009). Incorporation of sparsity allows much easier interpretation of the components and restores statistical consistency of the coefficients. Several other benefits are gained through sparsity including that it addresses the need - in substantive research - of selecting those variables that are important for further investigation (Rasmussen & Bro, 2012) and economic aspects associated to the cost of measuring (many) variables (d’Aspremont et al., 2005).

Sparseness of PCA weights and loadings can be obtained in multiple ways. They can be constrained with respect to the number of estimated non-zero elements, or penalty terms such as the lasso can be added to them. Several such constrained or penalized PCA formulations have been proposed in line with different objectives for PCA. Yet, whereas the different PCA problems can all be solved via the SVD, this does not hold for the different sparse PCA methods. Importantly, weights, loadings and right singular vectors are no longer mathematically equivalent to each other in the sparse setting. However, the difference among these structures has been largely overlooked, leading to questionable practices in the sparse PCA literature. First, most simulation studies in the literature restrict themselves to data generating schemes with sparseness residing in the right singular vectors or the loadings, instead of also incorporating models with sparseness in the weights. Second, it is a common practice to adopt the right singular vectors as initial values while local optimization procedures are employed for methods that impose sparsity on weights or loadings. These practices seem to ignore the fact that these quantities represent different model structures.

Our current paper aims to create awareness for the fact that weights and loadings are truly different model structures with different roles. We conduct a simulation study and employ empirical datasets in doing so. In our simulation study, the focus is on comparing the performance of sparse PCA methods in terms of criteria that matter for data analysis and explicitly taking into account that this difference between weights and loadings also resides at the level of the data generating model. Such a comparison has been made elsewhere (Guerra-Urzola et al., 2021; Van Deun et al., 2011) but with a somewhat different focus. The contribution of this work is to shed light on the performance of sparse loadings versus sparse weights in different data generating contexts by employing sparse PCA methods that are based on the same model formulation, allow exact control over the level of sparsity and account for local optima. Moreover, the difference between sparse loadings and sparse weights methods are also discussed in a more practical manner using empirical data.

The paper is arranged as follows: in the next section we detail formulations of PCA and sparse PCA. We highlight that the equality between weights, loadings and right singular vectors that exists within PCA is lost as the methods transition into sparse PCA. This is clearly illustrated by a toy example. In a simulation study we compare the performance of the sparse weights versus sparse loading methods under different data generation schemes and for different algorithm initialization strategies. By employing two different empirical datasets, we present the practical impact of the choice of sparse PCA formulation and the initialization strategy. The paper concludes with a discussion.

## Methods

After introducing the notation, we will first present the PCA decomposition and objective with special attention for the different roles played by component weights and loadings. We will highlight how the model structures (weights, loadings, singular vectors) are equal to one another. Then, we will discuss some commonly used sparse PCA methods that result from penalizing or constraining the PCA objective. It will be shown that the model structures are no longer subject to such equality within sparse PCA.

### Notation

Throughout the paper, vectors and matrices will be denoted by bold lowercase and bold uppercase letters respectively. Lowercase subscripts that run from 1 to the corresponding uppercase letters will be used for indexing: $i \in (1, 2, \ldots , I)$. *I*, *J* and *R* denote the total numbers of observation units, variables and components, respectively. For example, we will use $\textbf{X}$ to denote the data matrix, in which the *J* columns represent the variables and the *I* rows the observation units; note that the variables are assumed to be mean centered. Transposed vectors and matrices will be indicated by the superscript ^⊤^, therefore $I^{-1} \textbf{X}^{\top }\textbf{X}$ is the covariance matrix. The Frobenius norm for matrices is denoted as $\left\| . \right\| _F$ and the squared Frobenius norm $\Vert \textbf{X} \Vert ^2_F=\sum _{i,j}x^2_{i,j}$. Vector norms are defined as: $\left\| . \right\| _1$ for $\ell _1$ norm ($\left\| \textbf{x} \right\| _1 = \sum _i |x_i|$) and $\left\| . \right\| _2$ for $\ell _2$ norm ($\left\| \textbf{x} \right\| _2 = \sqrt{\sum _i x_i^2}$). $\textbf{Card}(.)$ indicates the cardinality of a matrix or a vector: this is the number of non-zero elements in the matrix or the vector. The addition of a subscript *R* to a matrix indicates the first *R* columns of the matrix.

### Principal component analysis

PCA has been presented in several ways that are mathematically equivalent (Jolliffe, 2002; Guerra-Urzola et al., 2021). The formulation incorporating both loadings and weights relies on the following decomposition of the data (Whittle, 1952; Gabriel, 1978; Wold et al., 1987):

1 $$\begin{aligned} \textbf{X}= & {} \textbf{T}_R \textbf{P}^{\top }_R+ \textbf{E}, \\ \nonumber \text {subject to } \textbf{P}^{\top }_R \textbf{P}_R= & {} \textbf{I}_R \text { and } \textbf{t}_r^{\top } \textbf{t}_{r'} = 0 \text { for } r \ne r', \end{aligned}$$

with $\textbf{T}_R$ ($I \times R$) denoting the principal component scores of the observation units and $\textbf{P}_R$ ($J \times R$) the *loadings* of the variables on the components. $\textbf{E}$ is the matrix of residuals which is assumed to be orthogonal to $\textbf{T}_R$. These parameters $\textbf{T}_R$ and $\textbf{P}_R$ are not unique; $\textbf{T}_R \textbf{A}$ and $\textbf{P}_R \textbf{A}^{-1}$ with an invertible matrix $\textbf{A}$ also suffice the model equation. The principal component scores are often written out as linear combination of the variables ($\textbf{T}_R = \textbf{XW}_R$) where $\textbf{W}_R$ ($J \times R$) matrix is referred to as *weights* which are understood analogously to regression weights in regression analysis.^1^ This can be explicitly expressed in the model: $\textbf{X} = \textbf{XW}_R \textbf{P}^{\top }_R+ \textbf{E}$ with the same constraints as in (1). To obtain the PCA decomposition of the data, a least squares criterion is used:

2 $$\begin{aligned} \hat{\textbf{T}}_R, \hat{\textbf{P}}_R= & {} \underset{{\textbf{T}_R, \textbf{P}_R}}{\mathrm {\arg \!\min }} \hspace{5.0pt}||\textbf{X} - \textbf{T}_R \textbf{P}_R^{\top } ||^2_{F} \\ \nonumber \text {subject to } \textbf{P}_R^{\top } \textbf{P}_R= & {} \textbf{I}_R \text { and } \textbf{t}_r^{\top } \textbf{t}_{r'} = 0 \text { for } r \ne r', \end{aligned}$$

Since $\textbf{T}_R = \textbf{XW}_R$, the solution for the weights $\hat{\textbf{W}}_R$ falls directly from the solution for the component scores $\hat{\textbf{T}}_R$ via least squares. In addition, the PCA objective (2) expresses the sum of squared errors between the observed data $\textbf{X}$ and its reconstruction $\textbf{T}_R \textbf{P}_R^{\top }$. Hence, the proportion of variance accounted for (VAF) by the estimated PCA model is computed by: $1 - ||\textbf{X} - \hat{\textbf{T}}_R \hat{\textbf{P}}_R^{\top } ||^2_{F} / ||\textbf{X} ||^2_F$. This VAF measure is commonly used as a measure of model fit for PCA or sparse PCA solutions, and adopted throughout the current paper.

***Mathematical equivalence of weights, loadings, and singular vectors***

As for the model (1), the problem in (2) is also not uniquely defined. Usually this issue is resolved by requiring a principal axis orientation of the principal components; they are found such that they successively explain maximum variance (Hotelling, 1933; Jolliffe, 2002). It is well known that the optimization problem in (2) can be solved via the singular value decomposition (SVD; see for example Jolliffe (2002)): Let $\mathbf {X = U S} \textbf{V}^{\top }$ with column orthogonal left singular vectors $\textbf{U}$ and right singular vectors $\textbf{V}$ ($\textbf{U}^{\top } \mathbf {U = I}_I$ and $\textbf{V}^{\top } \mathbf {V=I}_J$) and $\textbf{S}$ a $I \times J$ rectangular diagonal matrix with singular values in a decreasing order ($s_{11} \ge s_{22} \ge \ldots \ge 0$), then the rank *R* approximation $\mathbf {X \approx U}_R \textbf{S}_R \textbf{V}_R^{\top }$ is optimal in the least squares sense. Hence, adopting $\hat{\textbf{T}}_R = \textbf{U}_{R} \textbf{S}_{R}$, $\hat{\textbf{P}}_R = \textbf{V}_{R}$ and $\hat{\textbf{W}}_R = \textbf{V}_{R}$ provides the solution to the least squares problem in (2) under the set constraints. The weights and loadings are both provided by the right singular vectors and therefore are numerically equal to each other as they have the same value: $p_{jr}=v_{jr}=w_{jr}$.

***Conceptual difference between weights and loadings***

Despite weights and loadings being numerically equivalent, the two structures have different conceptual roles in the decomposition of data. The component weights $w_{jr}$ represent the weight that is given to a variable in the linear combination used to construct the component scores: $t_{ir}=\sum _j w_{jr}x_{ij}$. On the other hand, the loadings $p_{jr}$ represent the strength of association of the components with the observed variable: $x_{ij} \approx \sum _r t_{ir}p_{jr}$; note that this strength of association is not influenced by the other components because of their orthogonality. Under proper normalization constraints,^2^ the loadings are equal to the correlation between the observed variable and the component scores. Although both weight and loading matrices are equal to each other in the numerical sense, understanding their conceptual difference is important as their mathematical equivalence is lost for PCA decompositions relying on other constraints and optimization criteria than the ones presented in (1) and (2).

### Sparse principal component analysis

Sparse forms of PCA can be obtained by imposing sparseness either on the weights or the loadings in the PCA decomposition (1). Several sparse PCA methods have been proposed that rely on this idea (e.g., Zou et al., 2006; Shen and Huang, 2008; Witten et al., 2009; Erichson et al., 2020). Here, we focus on two well-known sparse PCA methods that rely on the least-squares approach to the decomposition, that extract all components simultaneously, and that allow exact control over the number of zero loadings or weights. These methods are SPCA (Zou et al., 2006) for the setting with sparse weights and USLPCA (Adachi & Trendafilov, 2016) for the setting with sparse loadings. Focusing on these two sparse PCA methods has the benefit that any observed differences in performance can be attributed to the choice for either sparse weights or loadings, ruling out alternative explanations such as algorithmic differences or differences in the level of sparsity.

***SPCA***

Zou et al. (2006) proposed the SPCA criterion, where an elastic net penalty is placed on the weights from the PCA objective (2) in which weights are explicitly written out:

3 $$\begin{aligned} (\hat{\textbf{W}}_R, \hat{\textbf{P}}_R)= & {} \underset{\textbf{W}_R, \textbf{P}_R}{\mathrm {\arg \!\min }} \hspace{5.0pt}\left\| \mathbf {X - XW}_R \textbf{P}_R^{\top } \right\| ^2_{F}\nonumber \\{} & {} + \lambda \sum _{r=1}^R \left\| \textbf{w}_r \right\| _1 + \lambda _2 \sum _{r=1}^R \left\| \textbf{w}_r \right\| _2^2 \nonumber \\{} & {} \text {subject to } \textbf{P}_R^{\top } \textbf{P}_R = \textbf{I}_R, \end{aligned}$$

with $\lambda \ge 0$ a tuning parameter for the lasso penalty; the effect of the penalty is that it shrinks the weights to zero, some/many of them even exactly so. In addition to the lasso, also a ridge penalty has been added. Its function is to obtain stable estimates in case of highly correlated predictors and to allow for more non-zero coefficients than *I* (in the setting with $J>I$).

The estimation of the weights and loadings is based on an alternating routine that updates the weights conditional upon the loadings and vice versa. The updating step of the sparse weights is based on the elastic net regression of the components on the variables. The SPCA procedure treats this problem with LARS-EN algorithm (Efron et al., 2004; Zou & Hastie, 2005) which allows the desired number of zero coefficients per component to be exactly specified in computing the weights.^3^ It is important to note that elastic net regression problems are known to have difficulties in identifying the true sparse model in the high-dimensional setting (Jia & Yu, 2010).

***USLPCA***

Sparsity can also be imposed to the loadings matrix in (2). Adachi and Trendafilov (2016) proposed a sparse PCA method by imposing a cardinality constraint on the loadings, leading to the USLPCA criterion:^4^

4 $$\begin{aligned} (\hat{\textbf{T}}_R, \hat{\textbf{P}}_R)= & {} \underset{\textbf{T}_R, \textbf{P}_R}{\mathrm {\arg \!\min }} \hspace{5.0pt}\left\| \mathbf {X - T}_R \textbf{P}_R^{\top } \right\| ^2_{F} \\ \nonumber \text {subject to } \textbf{T}_R^{\top } \textbf{T}_R= & {} \textbf{I}_R \text { and } \textbf{Card}(\textbf{p}_r) = k. \end{aligned}$$

USLPCA is also based on an alternating optimization procedure between the loadings and the component scores. The update of the loadings is a constrained univariate regression where each variable is regressed on each of the components. This implies that the estimates for the loadings do not suffer from stability issues in case of high correlations or high-dimensional data. Also, the estimation of the loadings easily allows the incorporation of a cardinality constraint in a computationally efficient way.

***Local optimality***

Unlike PCA formulations that have closed-form solutions based on SVD, iterative estimation procedures are adopted by the SPCA and USLPCA. As both methods are based on non-convex problems that are solved via an alternating procedure, the obtained solutions are prone to local optima. In their experiment of USLPCA, Adachi and Trendafilov (2016) reported that the method is sensitive to local optima characterized by solutions that are distant from the optimal solution. In order to aim for the global optimum, multiple random starting values should therefore be considered. Initializing these sparse PCA algorithms only with the right singular vectors can be problematic since it encourages the convergence to a local optimum near $\textbf{V}_R$.

***Loss of mathematical equivalence***

Under both sparse PCA formulations (3, 4), the equality among weights, loadings and right singular vectors is lost since SVD is no longer adopted as a direct solution. While SPCA finds sparse weights and non-sparse loadings, USLPCA finds sparse loadings. Weights from USLPCA, although not explicitly estimated, are non-sparse (they can be inferred by regressing the component scores $\textbf{T}_R$ on data $\textbf{X}$). Hence, within each sparse PCA formulation, the weights and loadings are different to each other and they are no longer equal to the right singular vectors of $\textbf{X}$. Across the two formulations, the weights and loadings estimated via the SPCA are different from the weights and loadings from the USLPCA.

#### Sparse PCA properties: toy example

In order to clearly illustrate the loss of equality among weights, loadings and right singular vectors for sparse PCA formulations, we make use of a toy example in this section. We created a $5 \times 3$ data matrix $\textbf{X}$ of rank two so the data can be perfectly reconstructed with $R=2$ components. A 2-component PCA model with sparse loadings underlies $\textbf{X}$ and therefore, the component scores $\textbf{T}$ are column-orthogonal ($\textbf{T}^{\top } \textbf{T} = \textbf{I}$) and the loadings $\textbf{P}$ are sparse as in (4):

5 $$\begin{aligned} \underset{\begin{array}{r}\\ [-10pt]{\textbf{X}} \end{array}}{ \left[ \begin{array}{rrr} 0.63 &{} 0.52 &{} 0.11 \\ -1.56 &{} -0.88 &{} 0.30 \\ 0.04 &{} 0.83 &{} 1.14 \\ 1.07 &{} 0.80 &{} 0.06 \\ -0.18 &{} -1.27 &{} -1.61 \\ \end{array}\right] }= & {} \underset{\begin{array}{r}\\ [-10pt]{\textbf{T}} \end{array}}{ \left[ \begin{array}{rr} 0.31 &{} 0.05 \\ -0.78 &{} 0.15 \\ 0.02 &{} 0.57 \\ 0.54 &{} 0.03 \\ -0.09 &{} -0.81 \\ \end{array}\right] }\\{} & {} \times \underset{\begin{array}{r}\\[3pt]{\textbf{P}^{\top }} \end{array}}{ \left[ \begin{array}{rrr} 2 &{} 0 \\ 1.41 &{} 1.41 \\ 0 &{} 2 \\ \end{array}\right] ^{\top } } \nonumber \end{aligned}$$

On this toy example dataset, we administered PCA, together with SPCA and USLPCA. Note that PCA weights and loadings are obtained by the right singular vectors. The two sparse PCA methods were applied such that one coefficient is returned sparse per component; this corresponds to the true sparse structure of the loading matrix in (5). The weights for USLPCA were calculated by regressing the estimated components on the variables. As aforementioned, these methods adopt the right singular vectors $\textbf{V}_R$ of $\textbf{X}$ by default as initial values for the iterative procedures. However, to account for the issue of local optima, a set of solutions stemming from 100 random initial values (with elements drawn from $\mathcal {U}(-1,1)$) were considered. The solution with the lowest value of the least squares loss was accepted as the final solution. We refer to the default approaches initialized by $\textbf{V}_R$ by SPCA-svd and USLPCA-svd, while the multistart versions are denoted by SPCA-multi and USLPCA-multi. Table 1 presents the solutions provided by the PCA and sparse PCA. The first column provides the loss values of each solution. Since the methods are characterized by different objective criteria, these values are only comparable across different initialization strategies within the same sparse PCA method. In addition, the last column concerns the model fit: VAF by each of the components ($1 - ||\textbf{X} - \hat{\textbf{t}}_r \hat{\textbf{p}}_r^{\top } ||^2_{F} / ||\textbf{X} ||^2_F$) and the total VAF ($1 - ||\textbf{X} - \hat{\textbf{T}}_R \hat{\textbf{P}}_R^{\top } ||^2_{F} / ||\textbf{X} ||^2_F$). The VAF values for the sparse PCA methods are computed in the same manner by replacing the PCA estimates with sparse PCA estimates.

**Table 1:** Solutions for the PCA and sparse PCA methods

Method	$\hat{{W}}_R$	$\hat{{P}}_R$	$\hat{{T}}_R$	*VAF*
PCA (2) loss 0	$$\left[ \begin{array}{rr} 0.50 &{} 0.71 \\ 0.71 &{} 0 \\ 0.50 &{} -0.71 \\ \end{array}\right] $$	$$\left[ \begin{array}{rr} 0.50 &{} 0.71 \\ 0.71 &{} 0 \\ 0.50 &{} -0.71 \\ \end{array}\right] $$	$$\left[ \begin{array}{rr} 0.73 &{} 0.37 \\ -1.25 &{} -1.32 \\ 1.18 &{} -0.78 \\ 1.14 &{} 0.71 \\ -1.80 &{} 1.01 \\ \end{array}\right] $$	$\text {vaf}_1 = 2/3, \ \text {vaf}_2 = 1/3, \ \text {vaf}_{\text {total}} = 1$
SPCA-svd loss 5.66	$$\left[ \begin{array}{rr} 0 &{} 0.71 \\ 1.41 &{} 0 \\ 0 &{} -0.71 \\ \end{array}\right] $$	$$\left[ \begin{array}{rr} 0.50 &{} 0.71 \\ 0.71 &{} 0 \\ 0.50 &{} -0.71 \\ \end{array}\right] $$	$$\left[ \begin{array}{rr} 0.73 &{} 0.37 \\ -1.25 &{} -1.32 \\ 1.18 &{} -0.78 \\ 1.14 &{} 0.71 \\ -1.80 &{} 1.01 \\ \end{array}\right] $$	$\text {vaf}_1 = 2/3, \ \text {vaf}_2 = 1/3, \ \text {vaf}_{\text {total}} = 1$
SPCA-multi loss 5.11	$$\left[ \begin{array}{rr} 0.83 &{} 0 \\ 0.56 &{} 0.02 \\ 0 &{} 1.14 \\ \end{array}\right] $$	$$\left[ \begin{array}{rr} 0.82 &{} -0.28 \\ 0.57 &{} 0.42 \\ -0.01 &{} 0.87 \\ \end{array}\right] $$	$$\left[ \begin{array}{rr} 0.81 &{} 0.13 \\ -1.78 &{} 0.33 \\ 0.50 &{} 1.32 \\ 1.34 &{} 0.09 \\ -0.86 &{} -1.87 \\ \end{array}\right] $$	$\text {vaf}_1 = 0.552, \ \text {vaf}_2 = 0.448, \ \text {vaf}_{\text {total}} = 1$
USLPCA-svd loss 1.17	$$\left[ \begin{array}{rr} 0.30 &{} 0.26 \\ 0.23 &{} -0.10 \\ 0.03 &{} -0.39 \\ \end{array}\right] $$	$$\left[ \begin{array}{rr} 1.85 &{} 0.77 \\ 1.85 &{} 0 \\ 0 &{} -1.85 \\ \end{array}\right] $$	$$\left[ \begin{array}{rr} 0.31 &{} 0.07 \\ -0.66 &{} -0.44 \\ 0.24 &{} -0.52 \\ 0.51 &{} 0.18 \\ -0.39 &{} 0.71 \\ \end{array}\right] $$	$\text {vaf}_1 = 0.569, \ \text {vaf}_2 = 1/3, \ \text {vaf}_{\text {total}} = 0.902$
USLPCA-multi loss 0	$$\left[ \begin{array}{rr} 0.38 &{} -0.13 \\ 0.18 &{} 0.18 \\ -0.13 &{} 0.38 \\ \end{array}\right] $$	$$\left[ \begin{array}{rr} 2 &{} 0 \\ 1.41 &{} 1.41 \\ 0 &{} 2 \\ \end{array}\right] $$	$$\left[ \begin{array}{rr} 0.31 &{} 0.05 \\ -0.78 &{} 0.15 \\ 0.02 &{} 0.57 \\ 0.54 &{} 0.03 \\ -0.09 &{} -0.81 \\ \end{array}\right] $$	$\text {vaf}_1 = 1/2, \ \text {vaf}_2 = 1/2, \ \text {vaf}_{\text {total}} = 1$

The VAF for each component and in total is indicated by $\mathrm {vaf_{1, 2}}$ and $\mathrm {vaf_{total}}$, respectively

We first study the solutions from PCA. As the loadings and weights are both derived from the right singular vectors $\textbf{V}_R$, the two are equal to each other. Now observing the sparse PCA solutions, we can notice that all of the sparse PCA formulations result in different solutions. The variance explained by each component and by both components collectively is also different across the formulations. The loss of equality among weights, loadings and right singular vectors is clear; within and between the sparse PCA formulations, the weight and loading matrices are not equal to each other, or to the right singular vectors $\textbf{V}_R$. A notable exception is the solution obtained by SPCA-svd which found loadings and component scores identical to those of PCA. This is because $\textbf{X}$ was generated from a 2-component PCA model without any noise and because SPCA-svd is initialized with the right singular vectors.^5^ However, as seen in the loss values, this is not the optimal solution in terms of the optimization criterion.

Our example also illustrates the role of initial values in sparse PCA formulations. Smaller loss was obtained by incorporating multiple starts for initialization. While the total amount of variance captured by the components was equal across the two initial value procedures for SPCA, the multistart approach explained more variance for USLPCA. Moreover, the coefficients attained by different initial value strategies show large discrepancies; this shows that neglecting the problem of local optima is consequential as it may result in lower VAF and inconsistency of the estimated weights and loadings. Unless the true model underlying the data is suspected to be characterized by sparse singular vectors, initializing the algorithm with only the right singular vectors may result in a suboptimal results.

#### Sparse PCA properties: some pitfalls

The loss of equality among weights, loadings and right singular vectors has a non-negligible consequence for simulation studies conducted to evaluate the sparse PCA methods; a data generating model characterized by sparse weights is disparate from another with sparse loadings or sparse singular vectors, and vice versa. It also has an important implication with respect to choice of the method in practical applications. This is evident in the toy example. The true sparse loadings in (5) were only recovered correctly by USLPCA-multi. PCA and SPCA, which target the right singular vectors and sparse weights respectively, were unable to recover the true sparse loading structure.

However, in the sparse PCA literature, models comprised of sparse loadings or sparse singular vectors have been predominantly employed for data generation, regardless of the structure (weights, loadings and right singular vectors) being sparsified by the method (e.g. Shen and Huang, 2008; Johnstone and Lu, 2009; Wang and Fan, 2017; Zou et al., 2006). The current literature has therefore largely overlooked the data generating model with sparse weights for simulation studies. In papers which propose a sparse PCA formulation with sparse loadings or sparse singular vectors, excluding the model with sparse weights can be seen as an incomprehensive practice. In other works that propose a sparse weights formulation, neglecting the model with sparse weights for data generation can be considered erroneous. A further complicating factor is that data generated with the sparse weights model poses a more difficult challenge for sparse PCA methods in retrieving the true parameters than the other two models. This implies that many of the results in the sparse PCA literature can be expected to be over-optimistic.

#### Data generating models

This section provides the data generating models (DGM) each comprised with sparse singular vectors, sparse loadings and sparse weights. We also discuss why the sparse weights model is more challenging to analyze than the other two models. Data from the model with sparse right singular vectors can be generated from the model $\textbf{X} = \textbf{U}_R \textbf{S}_R \textbf{V}_R^{\top } + \textbf{E}$ where the right singular vectors $\textbf{V}_R$ are sparse and column-orthogonal with norm equal one. This is equivalent to generating data from a multivariate normal distribution characterized by a zero mean vector and covariance matrix $\mathbf {\Sigma } = \textbf{V}_R \textbf{S}_R^2 \textbf{V}_R^{\top } + \mathbf {\Sigma }_E$^6^ where $\textbf{S}_R^2$ denotes the covariance matrix among components. Note that all off-diagonal elements are equal to zero as the component scores are orthogonal. This sparse singular vectors model is referred to as the ***spiked covariance model*** (Johnstone, 2001). With the right singular vectors defined sparse, this model simultaneously comprises sparse weights and sparse loadings ($\textbf{V}_R = \textbf{W}_R = \textbf{P}_R$). From the model $\textbf{X} = \textbf{T}_R \textbf{P}_R^{\top } + \textbf{E}$ with $\textbf{P}_R$ the sparse singular vectors, it follows that $\textbf{X} \textbf{P}_R = (\textbf{T}_R \textbf{P}_R^{\top } + \textbf{E}) \textbf{P}_R = \textbf{T}_R$, so $\textbf{P}_R$ indeed comprises the weights that make up the component scores $\textbf{T}_R$.

The DGM with sparse loadings (***sparse loadings model***)^7^ is derived from the PCA decomposition (1), in which the loadings are defined sparse: $\textbf{X} = \textbf{T}_R \textbf{P}^{\top }_R + \textbf{E}$. The USLPCA formulation (4) imposes this model. It is closely related with the spiked covariance model because it coincides with generating from the multivariate normal distribution with covariance matrix $\mathbf {\Sigma } = \textbf{P}_R \textbf{P}_R^{\top } + \mathbf {\Sigma }_E$. In fact, if $\textbf{P}_R$ is further constrained to be column-orthogonal, the sparse loadings model is equal to the spiked covariance model.

Lastly, to obtain the model with sparse weights (***sparse weights model***) the PCA decomposition with the weights written out is adopted ($\textbf{X} = \textbf{XW}_R \textbf{P}^{\top }_R+ \textbf{E}$) and sparsity is induced in the weights matrix. This model is implicitly assumed by the SPCA formulation (3). It does not coincide with sparse loadings or spiked covariance models. In comparison to the two models, the sparse weights model poses a much more complicated challenge for the sparse PCA methods for two reasons. First, the component scores $\textbf{XW}_R$ are post-multiplied by the loadings $\textbf{P}_R$ in constructing the data. This implies that the sparseness structure in the weights may not be clearly reflected in the observed data. Second, it suffers from the problem of indeterminacy; different $\textbf{W}$ matrices can lead to the same component scores $\textbf{XW}_R$.^8^ Appendix A provides an example illustrating this indeterminacy problem.

The sections above emphasized the difference between weights, loadings and singular vectors within sparse PCA. Models comprised with any of these sparse structures are also disparate from each other. In the following section, we evaluate common sparse PCA methods on the three different sparse PCA models to demonstrate the consequence of neglecting the difference between these structures and the models comprised of them.

## Simulation study

We present a critical assessment of the sparse PCA methods by taking into account 1) the three different data generating models characterized by sparse weights, sparse loadings and sparse singular vectors and 2) that the sparse PCA solutions resulting from SPCA and USLPCA are subject to the the problem of local optima. Each of the generated data sets is analyzed by each of the methods. Both the effectiveness of the methods at retrieving the true underlying model and at reconstructing the data are evaluated.

It is expected to be more difficult for the sparse PCA methods to reveal the underlying model if the data is generated from the sparse weights model, compared to the other two models. We anticipate that the initial value approaches would lead to different results, as demonstrated by the toy example. We also expect that the difference in performance between the SVD-based and multistart approach will be larger for data generated from the sparse weights model, due to the indeterminacy problem; since multiple different weights matrices can be viable solutions given the same component scores, the initial values would play a role in finding the solution that matches with the true parameters. With respect to the methods’ quality of capturing the variance in the data which can be quantified by the VAF measure, it is expected that the sparse weights method would perform better than the sparse loadings method. This is because high levels of sparseness in the loadings can result in all of the loadings corresponding to a variable being estimated as zero. This variable would not at all be accounted for by the model (all scores on the variable become equal to zero), resulting in small VAF. Lastly, with regards to retrieving the true parameters, because SPCA estimates sparse weights and USLPCA sparse loadings, one may expect the SPCA weights to better recover the true weights than the USLPCA loadings and also the USLPCA loadings to better recover the true loadings than the SPCA weights. For the recovery of the true loadings, this is a reasonable expectation. However, given the indeterminacy of the weights and when these are generated under the spiked covariance model, it is more reasonable to expect the USLPCA loadings to better recover the sparse weights (as in this setting loadings and weights are equal yet estimation of the loadings is stable unlike the weights). Nevertheless, it is in general difficult to hold a clear expectation about SPCA and elastic net as they suffer from problems such as indeterminacy under high dimensionality.

**Algorithm 1:**
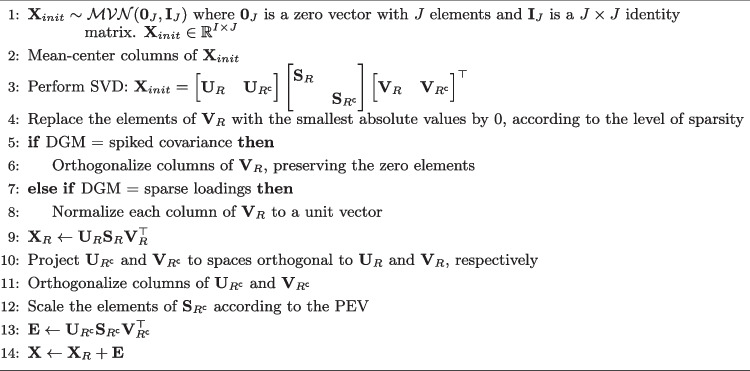
Spiked covariance and sparse loadings data generation.

### Design and procedure

Along with the three DGMs, various other data characteristics of the datasets were also manipulated in order to study the interaction between sparse PCA methods, data characteristics and DGMs. Fixing the number of components *R* to two, we generated datasets via the design below. For each manipulated design factor, the levels are provided between square brackets.

***Study design***

1. Data generating model (DGM): [Spiked covariance], [Sparse loadings], [Sparse weights]
2. Dimensions of $\textbf{X}$ ($I \times J$): [Low-dimensional ($100 \times 50$)], [High-dimensional ($100 \times 500$)]
3. Level of sparsity in the coefficients matrix: [90%], [50%]
4. Proportion of error variance in $\textbf{X}$ (PEV): [0%], [10%], [50%]

The following provides the scheme used to generate the data from spiked covariance and sparse loadings models. We adapted the setups devised in Johnstone (2001) (spiked covariance) and in Zou et al. (2006) (sparse loadings).

$\textbf{U}_R$ and $\textbf{V}_R$ refer to the first *R* columns of $\textbf{U}$ and $\textbf{V}$ (left and right singular vectors), whereas $\textbf{U}_{R^\textsf{c}}$ and $\textbf{V}_{R^\textsf{c}}$ refer to the remaining $(J-R)$ columns, respectively. Similarly, $\textbf{S}_R$ and $\textbf{S}_{R^\textsf{c}}$ are the first $R \times R$ submatrix and the remaining $(J-R) \times (J-R)$ submatrix of $\textbf{S}$ (diagonal matrix with singular values), respectively. By relying on the SVD formulation, the model part ($\textbf{X}_R = \textbf{U}_R \textbf{S}_R \textbf{V}_R^{\top }$) and the error part ($\textbf{E} = \textbf{U}_{R^\textsf{c}} \textbf{S}_{R^\textsf{c}} \textbf{V}_{R^\textsf{c}}^{\top }$) of the final data matrix $\textbf{X}$ can be defined in an uncorrelated manner. $\textbf{S}_{R^\textsf{c}}$ is scaled such that the ratio between $\Vert \textbf{X}_R\Vert ^2_F$ and $\Vert \textbf{E} \Vert ^2_F$ reflects the PEV condition. Additionally, for the sparse loadings model, the true component scores matrix and the loadings matrix are defined by the following: $\textbf{T}_R = \textbf{U}_R$ and $\textbf{P}_R = \textbf{V}_R \textbf{S}_R$. The setup used to generate data according to the sparse weights model is provided in the following. Similar setups have been used in the literature (de Schipper and Van Deun 2018; Van Deun et al. 2011; Guerra-Urzola et al. 2021).

**Algorithm 2:**
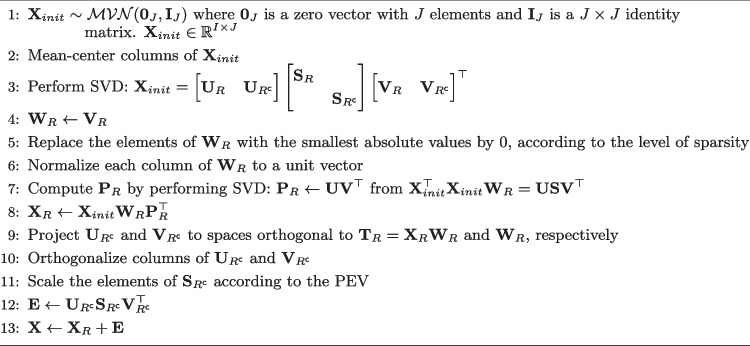
Sparse weights data generation.

$\textbf{E}$ is defined in the same manner as for Algorithm 1; via the SVD formulation, it is ensured that the model part and the error part are uncorrelated. The non-sparse loadings matrix $\textbf{P}_R$ is computed by solving the least squares problem with the orthonormality constraint $\textbf{P}^{\top }_R \textbf{P}_R = \textbf{I}_R$. The solution is given by $\textbf{P}_R = \textbf{U} \textbf{V}^{\top }$, where $\textbf{U}$ and $\textbf{V}$ are left and right singular vectors of $\textbf{X}_{init}^{\top } \textbf{X}_{init} \textbf{W}_R$ (ten Berge, 1993). This closed-form solution is used in several sparse weights estimation methods where the loadings matrix is constrained to be orthonormal, including the SPCA algorithm Zou et al. (2006).

For all three DGMs, the initial matrix $\textbf{X}_{init}$ can also be generated with correlation between the variables instead of using the diagonal covariance matrix. The results obtained from data generated with uncorrelated $\textbf{X}_{init}$ variables are very similar to the results from correlated $\textbf{X}_{init}$ matrix, which are reported in the Appendix B.

Fully crossing these factors provided in the study design resulted in $3 \times 2 \times 2 \times 3 = 36$ conditions, and 50 datasets were generated through the above schemes according to each condition. For each of the 1800 datasets, 4 analysis methods which resulted from crossing the following factors were administered.

***Analysis methods***

1. Sparse PCA method: [SPCA (sparse weights)], [USLPCA (sparse loadings)]
2. Initial value approach: [SVD-based], [Multistart]

The SPCA algorithm implemented in the R package ‘elasticnet’ was slightly adapted such that the algorithm can be initiated with starting values other than the right singular vectors. For the USLPCA procedure we employed our own R implementation. Both SPCA and USLPCA allow to specify the number of desired zero elements in the estimated coefficient matrix. Therefore the information of the true level of sparsity was provided as an input. The right singular vectors $\textbf{V}_R$ of the data $\textbf{X}$ were used as the SVD-based initial values. For the multistart approach, a set of the right singular vectors of $\textbf{X}$ and 19 other sets of randomly drawn values from uniform distribution $\mathcal {U}(-1,1)$ were incorporated as initial values. Each set was employed separately for estimation and the solution with minimum loss value was selected as the final solution of the multistart approach. In the following section, the four methods are referred to as SPCA-svd, SPCA-multi, USLPCA-svd and USLPCA-multi, respectively.

**Fig. 1:**
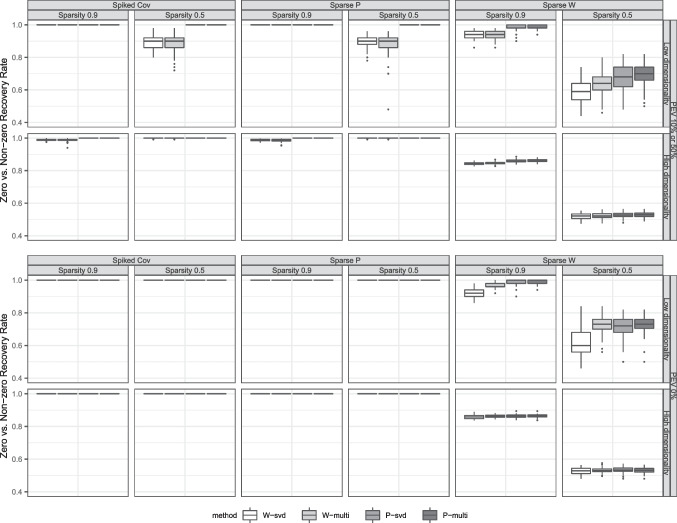
Box plots of zero versus non-zero recovery rate. The different columns correspond to the different DGM schemes and sparsity. The rows differ in the level of PEV and whether the data are low- or high-dimensional. The two top rows refer to results concerning datasets in which the defined components do not fully account for the variance in the data (error variance added on top of the DGM), while the bottom rows refer to datasets generated without any error variance

To examine the performance of the 4 methods with respect to retrieving the true model parameters and to reconstructing the data, three evaluation criteria were adopted:

***Evaluation criteria***

1. Zero versus non-zero recovery rate: the number of coefficients correctly estimated as zero or non-zero elements, divided by the total number of coefficients.
2. Component scores congruence: Tucker congruence computed between the estimated and the true component scores.
3. Proportion of variance accounted for (VAF) by the derived components.

The zero versus non-zero recovery rate is always calculated between the quantity being defined sparse in the true model and the quantity being estimated sparse by the method. For example, when USLPCA is used to analyze a dataset generated from the sparse weights model, the true sparse weights defined is compared against the sparse loadings estimated by USLPCA.

The congruence between the true component scores $\textbf{T}_R^{true}$ and the estimated scores $\hat{\textbf{T}}_R$ is measured by the Tucker congruence statistic $\phi$ which is defined as:

6 $$\begin{aligned} \phi = \frac{\text {vec}(\textbf{T}_R^{true})^{\top } \text {vec}(\hat{\textbf{T}}_R)}{\sqrt{(\text {vec}(\textbf{T}_R^{true})^{\top } \text {vec}(\textbf{T}_R^{true})) (\text {vec}(\hat{\textbf{T}}_R)^{\top } \text {vec}(\hat{\textbf{T}}_R))}}. \end{aligned}$$

**Fig. 2:**
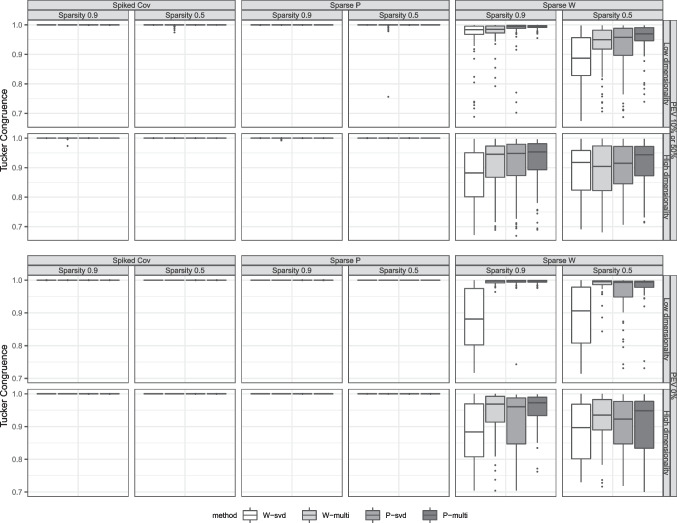
Box plots of component scores congruence. The different columns correspond to the different DGM schemes and sparsity. The rows differ in the level of PEV and whether the data are low- or high-dimensional. The two top rows refer to results concerning datasets in which the defined components do not fully account for the variance in the data (error variance added on top of the DGM), while the bottom rows refer to datasets generated without any error variance

### Results

#### Zero versus non-zero recovery rate

Figure 1 shows the boxplots of the zero versus non-zero recovery rate. We separated the results according to whether the datasets were generated such that the defined underlying components completely account for the variance in the data (conditions with zero error variance, in the two bottom rows) or not (10% or 50% of error variance, top rows). The figure first shows that datasets generated from the sparse weights model resulted in a much lower quality of zero versus non-zero recovery than the other two DGMs. Even when the defined components completely explain the variance in the data, the methods resulted in poor performance under the sparse weights model. In contrast, all of the methods resulted in perfect recovery of the coefficients under the spiked covariance and the sparse loadings generation schemes when no error variance was added on top of the true model structure. With respect to the performance of sparse weights versus sparse loadings methods, USLPCA showed an overall higher recovery rate than SPCA. Concerning the initial value approaches, the multistart approach yielded a higher recovery rate than SVD-based initial values.

**Fig. 3:**
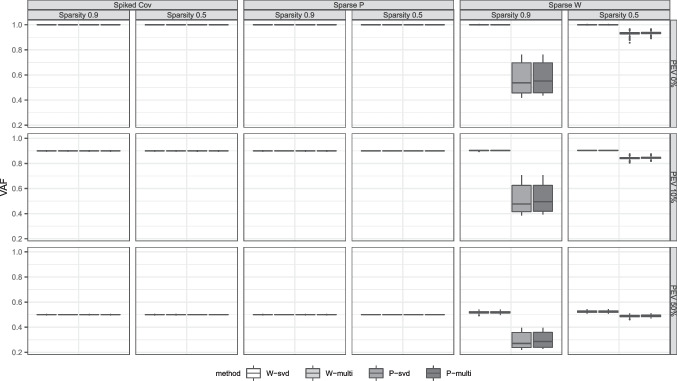
Box plots of proportion of variance accounted for. The different columns correspond to the different DGM schemes and sparsity. The rows differ in the level of PEV by the different components

#### Component scores congruence

Figure 2 displays the results on the component scores congruence laid out in the same format as Fig. 1. The results are largely in agreement with those concerning the zero versus non-zero recovery rate of the coefficients; the DGMs other than the sparse weights model led to good performance, while the methods struggled on datasets generated from the sparse weights model. Concerning the spiked covariance model and the sparse loadings model, all of the methods performed nearly perfectly in retrieving the component scores, except for SPCA which led to some poor outlying results when error variance was added on top of the defined DGM.

Although the methods performed poorly for the sparse weights model compared to the other models, it can be seen that the median Tucker congruence of the methods were in most cases above 0.9. Components with a congruence value in between 0.85 and 0.94 are often seen as fairly similar (Lorenzo-Seva & Ten Berge, 2006). Despite the low zero versus non-zero recovery of true weights shown in Fig. 1, the component scores were recovered quite well by the methods. This hints back at the indeterminacy problem of the sparse weights model. Although the component scores are well recovered, it is difficult for the methods to retrieve the true weights since there are multiple different weights matrices that can construct very similar component scores. Lastly, the impact of the starting values is also seen; the multistart approach yielded better results than initializing the methods with SVD solutions.

#### Proportion of variance accounted for (VAF)

While the two evaluation criteria above reflect the behaviour of the methods with regards to retrieving the underlying model parameters, VAF pertains to the degree to which the methods restore the observed data. Figure 3 presents these results. Dimensionality did not lead to differential results for VAF thus aggregated results are presented. The boxplots show that the most impactful factor for reconstruction quality is the proportion of additional error variance in the observed dataset on top of the true components. As more noise is added to the DGM, the methods exhibit smaller VAF. It is interesting to see that across all of the conditions, the approach of initializing the algorithm did not exert an influence, unlike in the other evaluation criteria above. While all four methods performed comparably for the spiked covariance model and the sparse loadings model, the USLPCA methods underperformed compared to SPCA methods for the datasets generated with sparse weights. Moreover, with this exception of USLPCA being administered to data from the sparse weights model, the methods have succeeded in capturing the correct proportion of variance accounted for by the true components. For the condition with 50% of error variance, the methods have even explained slightly more variance than the true proportion of variance. This can be seen as a case of overfitting; on top of capturing the variance defined by the true underlying components, the methods seem to also explain a small amount of error variance.

### Discussion of the results

The results from the simulation study are mainly in line with our expectations. While the sparse PCA methods resulted in near-optimal performance in finding back the true parameters under the spiked covariance and sparse loadings models, they showed much poorer performance under the sparse weights model. The methods struggled particularly with respect to recovering the true weights. This finding is quite alarming because it implies that the conclusions being drawn from the current sparse PCA literature dominated by the spiked covariance and spiked loadings models may be over-optimistic.

Our results also clearly illustrate the impact of initial values for these sparse PCA methods. For both evaluation criteria of zero versus non-zero recovery rate and component scores congruence, the multistart approach resulted in better performance than starting with the right singular vectors. In particular, the difference in the performance between the initial value approaches was large under the sparse weights model. Considering that the true model underlying data is unknown in practice, it is important to adopt multiple starting values for sparse PCA methods that are characterized by local optima, such as SPCA and USLPCA in our simulation study.

Finally, our simulation study highlights the difference between the results obtained by sparse loadings versus sparse weights methods. In our toy example above, it was shown that the methods lead to different estimates and therefore different insights about the same dataset. The simulation study extends this difference towards performance of the methods. It was shown that SPCA with sparse weights was poorer at zero versus non-zero recovery of parameters under all data generation schemes than USLPCA with sparse loadings. SPCA resulted in underperformance compared to USLPCA even when the data was generated from the sparse weights model. However, SPCA was better suited at deriving components that explain a large amount of variance in the data if the data were generated under a sparse weights model and performed nearly as well as USLPCA under the sparse spiked covariance and loading scheme. This finding implies that the methods must be chosen carefully in practice.

## Empirical application

In this section we further illustrate the practical impact of the choice between the sparse weights and sparse loadings methods coupled with initialization strategies in an empirical setting.

### Big five dataset IPIP-NEO-120

We adopted a dataset comprised of 120 items of the IPIP-NEO-120 questionnaire. The IPIP-NEO-120 scale is a set of public domain items designed to measure the Big Five personality traits (Goldberg et al., 1999); each of the personality traits is measured by 24 items. We downloaded the raw data collected with the questionnaire which is publicly available from the online repository: https://osf.io/wxvth/ (Johnson, 2018). The raw data was collected via a large internet survey conducted for the construction of the questionnaire (Johnson, 2014) in which 619150 subjects participated, and we selected the first 1000 observations of the data for our sparse PCA analysis to ease the computational burden.

As the questionnaire measures five underlying constructs, we fixed the number of components to be five. Also following the design of the questionnaire, the level of sparsity was determined such that 24 non-zero coefficients are estimated per component. In order to examine the impact of choosing between the sparse weights and sparse loadings methods and the initial value approaches, we applied the methods used in the simulation study: SPCA-svd, SPCA-multi, USLPCA-svd and USLPCA-multi.

The weights from SPCA methods and the loadings from USLPCA methods were inspected to interpret the found components. Table 2 presents the numbers of items designed for each of the personality traits which have a non-zero coefficient for each of the components.

**Table 2:** Big Five dataset: sparse PCA methods with 24 non-zero coefficients per component (O: openness, C: conscientiousness, E: extraversion, A: agreeableness, N: neuroticism)

	t1	t2	t3	t4	t5
SPCA-svd weights					
O	20	2	1	1	0
C	0	18	0	1	4
E	0	4	19	4	1
A	4	0	0	17	1
N	0	0	4	1	18
SPCA-multi weights					
O	20	2	1	1	0
C	0	18	0	1	4
E	0	4	19	4	1
A	4	0	0	17	1
N	0	0	4	1	18
USLPCA-svd loadings					
O	19	0	1	4	0
C	0	16	5	1	0
E	1	7	11	0	6
A	3	0	2	19	0
N	1	1	5	0	18
USLPCA-multi loadings					
O	19	3	1	1	1
C	0	18	1	1	0
E	0	3	17	4	2
A	5	0	0	14	3
N	0	0	5	4	18

The columns indicate each component while the rows indicate each personality trait

Table 2 presents the similarities between the models constructed by the sparse weights and the sparse loadings methods. For all four methods, a majority of the non-zero coefficients on each component correspond to items that measure the same personality trait. As the items in the scale operationalize the five-factor model of personality traits (FFM; McCrae and Costa Jr (2008)), the models found by the four methods seem to nicely reflect the true model behind the observed items. This goes together with the results in our simulation study where the sparse weights and the sparse loadings methods were both capable at finding the underlying structure when low-dimensional data was generated from a spiked covariance or sparse loadings structure. The FFM resembles these models as the subsets of 24 items load on the five factors.

The table also shows the role of initial values in these sparse PCA methods. While all of the non-zero weights found by the two SPCA methods corresponded to each other, different initial values have led to different models being constructed for USLPCA. The third component from USLPCA-svd can be interpreted as a mix between extraversion, conscientiousness and neuroticism, while the third component from USLPCA-multi is less diffuse. This demonstrates the impact of initial value strategies in practice where sparse PCA models are used.

Finally, the findings from the simulation study regarding the proportion of variance explained by the sparse PCA methods are also echoed with this dataset. SPCA methods accounted for more variance than USLPCA methods. SPCA-svd and SPCA-multi models explained 32.3% and 32.3% of variance in the data while USLPCA-svd and USLPCA-multi fell short at 25.9% and 27.3%.

### Autism gene expression data

This dataset concerns gene expression profiles of three groups: 6 male subjects with autism caused by fragile X syndrome (FMR1-FM), 7 male subjects with autism caused by inherited duplication of 15q11-q13 (dup15q) and 14 non-autistic control male subjects (Nishimura et al., 2007).^9^ The dataset consisted of 43893 probe sets measuring the transcription rates of about 20 thousand genes for each subject. In the original publication, the authors selected a subset of the probes that are important at discerning the three groups by inspecting the *p* values derived by univariate ANOVA. The authors continued on by conducting PCA with 3 components on this subset to explore the mechanism underlying the probes.

Building on the original publication, we also conducted ANOVA to obtain a subset of probes which are strongly related with the group membership of the subjects. Prior to our analysis, each column of the dataset was mean-centered and standardized to unit variance. ANOVA resulted in 107 probes with *p* values smaller than 0.05. As our current paper discusses sparse PCA, we also sampled 1000 other ‘redundant’ probes among the probes with *p* values greater than 0.5 and constructed a dataset of 1107 probes. We did not use the entire set of variables to reduce the computational burden. The four methods SPCA-svd, SPCA-multi, USLPCA-svd and USPLCA-multi were then administered, in order to study these differences between the methods in providing the sparse components comprised of important and redundant probes.

The number of components was fixed at 3 as in Nishimura et al. (2007). As there are 107 important probes, we administered the methods with 107 non-zero coefficients per component and studied the returned coefficients.^10^ Unlike the Big Five dataset above, the data generating model underlying the autism gene expression dataset is ambiguous; the nature of the mechanisms governing the gene expressions is unknown. Since it is not possible to compare the retrieved sparse PCA models with the true structure, we compared the results among themselves. Table 3 presents the proportion of non-zero coefficients that correspond across each pair of the four methods (above) and the Tucker congruence values between the component scores computed by the methods (below).

**Table 3:** Autism dataset

	SPCA-svd	SPCA-multi	USLPCA-svd
Proportion of corresponding non-zero coefficients			
SPCA-multi	0.283		
USLPCA-svd	0.461	0.461	
USLPCA-multi	0.305	0.452	0.573
Component scores Tucker congruence			
SPCA-multi	0.685		
USLPCA-svd	0.818	0.796	
USLPCA-multi	0.629	0.780	0.728

Above: proportion of corresponding non-zero coefficients out of the total 321 (107 non-zero coefficients $\times 3$ components). Below: Tucker congruence between the component scores

Table 3 conveys that the models derived by the four methods are quite different. Within the same formulation of SPCA, only 28.3% of the non-zero weights found by the multistart approach were also found by employing the starting values based on SVD. Likewise, 57.3% of the non-zero loadings from the two starting value approaches corresponded to each other for USLPCA. This proportion of corresponding non-zero coeffcients is also low when comparing weights from the SPCA methods against loadings from the USLPCA methods. On the other hand, the congruence values among the components were higher; although largely different sets of variables were picked up by the different methods, the estimated components ended up rather correlated. This is in line with our results from the simulation study where the congruence between the estimated and the true components were high for the sparse weights model, despite its low zero versus non-zero recovery rate. Nevertheless, the congruence scores between the methods concerning the current autism dataset were all lower than 0.85 which is a value expected for fairly similar components (Lorenzo-Seva & Ten Berge, 2006). Altogether, these results imply that the four methods would provide components that are understood as being different from each other. They reiterate the findings from the simulation study that showed different performances of the methods depending on the sparse PCA formulation and the initial value strategies of choice.

With respect to the proportion of explained variance, SPCA-svd and SPCA-multi both recovered 36.6% of the data. On the contrary, USLPCA-svd and USLPCA-multi resulted in 16.3% and 16.4%. Like in the simulation study, the choice between sparse weights and loadings led to a considerable difference in the amount of variance explained.

## Conclusion

The contribution provided by this paper concerns an important warning towards the difference between weights and loadings and its implications. Section 2 discussed the theoretical difference. It was shown that the weights and the loadings are not equal to each other or to the right singular vectors within sparse PCA, making it important for the quantities to be distinguished carefully. We pointed out that this loss of equality between the quantities is not well reflected in research employing sparse PCA. Namely, a vast majority of simulation studies confine themselves to data generating models characterized by sparse singular vectors or sparse loadings, and most sparse PCA methods initialize the algorithms with SVD solutions. Through a simulation study, we demonstrated that such practices paint a wrong picture about the performance of sparse PCA methods. In fact, reported simulation studies have been dominated by the spiked covariance and sparse loadings model, also to study methods that estimate sparse weights. Based on such studies, over-optimistic conclusions have been drawn about the performance of the sparse weights method in recovering the underlying zero-nonzero structure of the data. A related issue is the combination of generating data under a sparse SVD structure in combination with SVD based starting values; as shown in the toy example, also the sparse loadings method suffers from recovering the underlying sparseness structure when sparseness does not reside in singular vectors. The importance of using methods that implement a multi-start initialization strategy was discussed and shown throughout the paper.

Our paper also touches upon the issue of choosing between sparse weights and sparse loadings PCA. In practice, in making an informed choice between them, we consider the research aim as the first aspect to take account of. If it is expected that the variables are associated to a few underlying components and one wishes to find a sparse representation of the relationships between the components and the variables, sparse loadings PCA is suitable. On the other hand, as reported in our simulation study, if one aims to derive summary scores that account for a great amount of variance in the variables, sparse weights PCA should be the choice.

Besides the aim of the analysis (summarizing versus exploring the component-variable associations), often domain-related beliefs regarding the data may determine the choice between the sparse loadings and sparse weights model. For example, psychological scales are often constructed according to a sparse loadings model in the sense that the variables (items) are designed to measure particular latent constructs: the variables are reflective indicators of a latent variable (Hwang et al., 2021). Personality questionnaire data such as one provided in Section 4 would serve as an example, or measurements of the construct IQ.

On the other hand, the sparse weights model may be considered appropriate when the interest is to measure indices of observable constructs (e.g., poverty index) in a context where such indices are not yet known. This is for example of particular interest for the construction of genetic risk scores. Data originating from sparse weights models would be comprised of variables that linearly combine into a component. Economic data containing variables that combine and form an economic index is another example; education level, income, occupation and other variables link up together to form socioeconomic status (e.g. Hauser and Warren, 1997; Thomson, 2018). Here, the observed variables form the component and are therefore often referred to as composite indicators Hwang et al. (2021).

Our findings from the datasets generated from the sparse weights model can appear counterintuitive to existing literature concerning the consistency of sparse PCA methods. In the data generation, these studies have employed the spiked covariance model. Our findings are therefore in agreement with them, as the sparse PCA methods were very good at correctly revealing the spiked covariance model or the sparse loadings model. The contribution of our work is showcasing that when other plausible sparse PCA models underlie data, the sparse PCA methods may not be as optimal in recovering the data generating model. Note that this does not imply that these previous studies are irrelevant as they indeed demonstrate the effectiveness of sparse PCA in the high-dimensionality-low-sample-size setting where PCA is known to perform poorly.

We conclude with a plea. Simulation studies should not be restricted to sparse singular vectors or sparse loadings structures, as (A) different models may underlie data in practice and (B) certain sparse PCA formulations impose a model structure that do not match these structures. Also, multiple starting values should be considered along with the solutions of SVD. Many sparse PCA formulations are characterized by non-convex problems, and limiting the starting values at the SVD solutions can push the methods to converge into a local optimum. The loss of equality between weights, loadings and right singular vectors in the context of sparse PCA should be carefully acknowledged.

## Figures and Tables

**Fig. 4:**
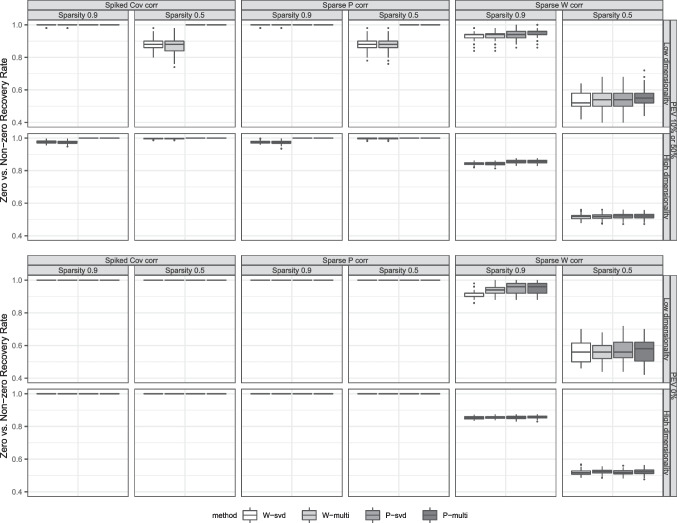
Box plots of zero versus non-zero recovery rate. The different columns correspond to the different DGM schemes and sparsity. The rows differ in the level of PEV and whether the data are low- or high-dimensional. The two top rows refer to results concerning datasets in which the defined components do not fully account for the variance in the data (error variance added on top of the DGM), while the bottom rows refer to datasets generated without any error variance

**Fig. 5:**
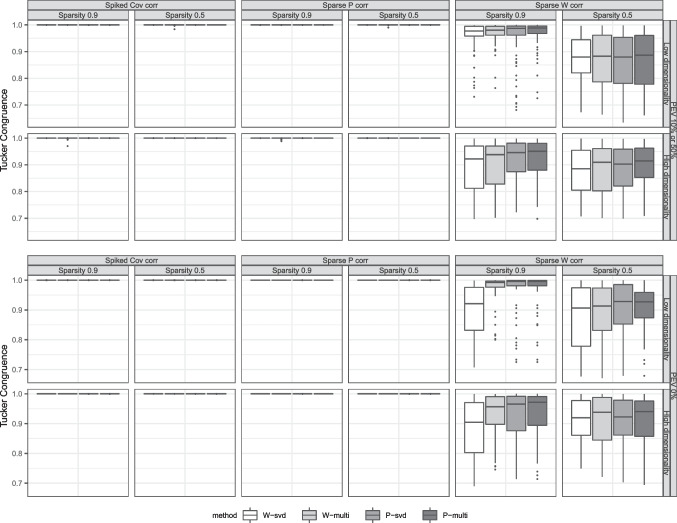
Box plots of component scores congruence. The different columns correspond to the different DGM schemes and sparsity. The rows differ in the level of PEV and whether the data are low- or high-dimensional. The two top rows refer to results concerning datasets in which the defined components do not fully account for the variance in the data (error variance added on top of the DGM), while the bottom rows refer to datasets generated without any error variance

**Fig. 6:**
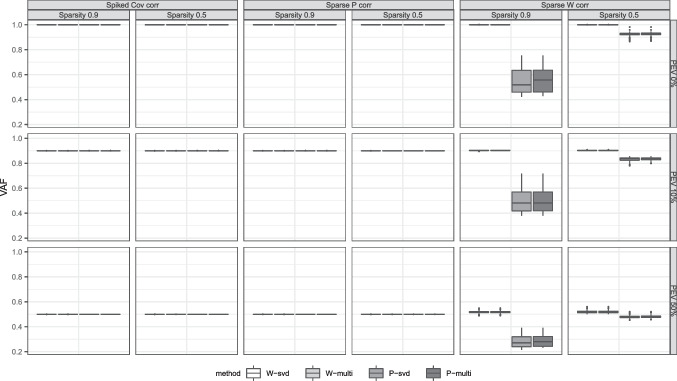
Box plots of proportion of variance accounted for. The different columns correspond to the different DGM schemes and sparsity. The rows differ in the level of PEV by the different components

**Algorithm 3:**
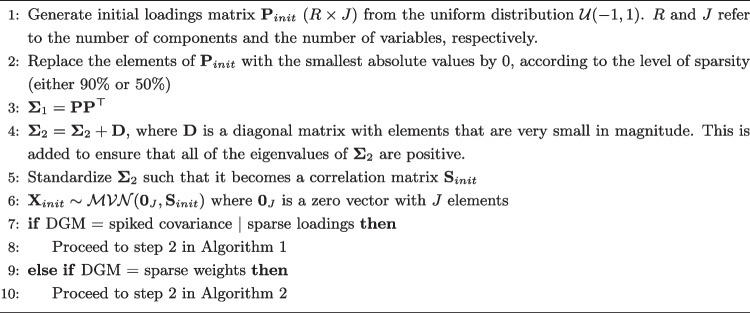
Correlated initial data matrix generation.

## Data Availability

Code used to generate the results reported in the manuscript is available on Github: https://github.com/soogs/Sparse-PCA-Critical-Assessment. None of the experiments were pre-registered. The IPIP NEO big five personality data is publicly available from the online repository: https://osf.io/wxvth/ (Johnson, 2018). The autism dataset is available from the NCBI GEO database with accession code GSE7329.

## Appendix A: Sparse weights indeterminacy problem

Deriving the true sparse weights from the data is a difficult task because of the indeterminacy problem of the weights; different $\textbf{W}_R$ matrices can construct the same component scores $\textbf{XW}_R$. For high-dimensional data, this implies that different sets of variables can be combined to lead to the same component scores. We provide a small example conveying the problem in this section.

We have generated a small data matrix $\textbf{X}$ of size $3 \times 5$ from a multivariate normal distribution characterized by a zero vector of length 5 for the mean and a $5 \times 5$ identity matrix for the covariance. After centering and standardizing the variables, we can extract one component that captures the largest amount of variance by performing PCA, which provides the following weights and component scores:

7 $$\begin{aligned} \underset{\begin{array}{c}\\[2.5pt]\textrm{X}\end{array}}{ \left[ \begin{array}{rrrrr} -1.37 &{} -1.40 &{} -0.99 &{} -1.36 &{} -1.20 \\ 0.37 &{} 0.52 &{} 1.37 &{} 1.01 &{} 1.25 \\ 0.99 &{} 0.88 &{} -0.37 &{} 0.35 &{} -0.05 \\ \end{array}\right] } \times \underset{\begin{array}{c}\\ [-8.5pt]\hat{\textrm{w}} \end{array}}{ \left[ \begin{array}{r} 0.43 \\ 0.45 \\ 0.41 \\ 0.48 \\ 0.46 \\ \end{array}\right] } = \underset{\begin{array}{c}\\[2.4pt]\hat{\textrm{t}} \end{array}}{ \left[ \begin{array}{r} -2.83 \\ 2.02 \\ 0.81 \\ \end{array}\right] } \end{aligned}$$

The indeterminacy problem is that there are many solutions for $\textbf{w}$ different from the one in the above equation that yield the same component scores. This means that when administering a sparse weights PCA method, any linear combination of 3 out of the 5 variables can lead to the same component scores. Below are two such weights:

8 $$\begin{aligned} \underset{\begin{array}{c}\\[2.5pt]\textrm{X}\end{array}}{ \left[ \begin{array}{rrrrr} -1.37 &{} -1.40 &{} -0.99 &{} -1.36 &{} -1.20 \\ 0.37 &{} 0.52 &{} 1.37 &{} 1.01 &{} 1.25 \\ 0.99 &{} 0.88 &{} -0.37 &{} 0.35 &{} -0.05 \\ \end{array}\right] } \times \underset{\begin{array}{c}\\ [-8.5pt]\hat{\textrm{w}} \end{array}}{ \left[ \begin{array}{r} 0 \\ 0 \\ -0.49 \\ 1.90 \\ 0.62 \\ \end{array}\right] } = \underset{\begin{array}{c}\\[2.7pt]\hat{\textrm{t}} \end{array}}{ \left[ \begin{array}{r} -2.83 \\ 2.02 \\ 0.81 \\ \end{array}\right] } \end{aligned}$$

9 $$\begin{aligned} \underset{\begin{array}{c}\\[2.5pt]\textrm{X} \end{array}}{ \left[ \begin{array}{rrrrr} -1.37 &{} -1.40 &{} -0.99 &{} -1.36 &{} -1.20 \\ 0.37 &{} 0.52 &{} 1.37 &{} 1.01 &{} 1.25 \\ 0.99 &{} 0.88 &{} -0.37 &{} 0.35 &{} -0.05 \\ \end{array}\right] } \times \underset{\begin{array}{c}\\ [-8.5pt]\hat{\textrm{w}} \end{array}}{ \left[ \begin{array}{r} 0.60 \\ 0.69 \\ 1.05 \\ 0 \\ 0 \\ \end{array}\right] } = \underset{\begin{array}{c}\\[2.7pt]\hat{\textrm{t}} \end{array}}{ \left[ \begin{array}{r} -2.83 \\ 2.02 \\ 0.81 \\ \end{array}\right] } \end{aligned}$$

In a high-dimensional setting where the number of observations is smaller than the number of variables, retrieving the correct underlying sparse weights from many possible solutions can therefore be a complicated task. Different sets of variables can combine into the same component scores.

## Appendix B: Simulation study with data generation where initial data matrix is generated with correlation

This section presents a simulation study employing data generation schemes that start off with an initial data matrix with correlated variables. Section 3.1 provides the motivation behind these additional schemes.

### Design and procedure

Fixing the number of components *R* to two, we generated datasets via the following design. The levels for the data generating model factor are the only difference from the study design presented in Section 3.1.

***Study design***

1. Data generating model (DGM): [Spiked covariance (correlated)], [Sparse loadings (correlated)], [Sparse weights (correlated)]
2. Dimensions of $\textbf{X}$ ($I \times J$): [Low-dimensional ($100 \times 50$)], [High-dimensional ($100 \times 500$)]
3. Level of sparsity in the coefficients matrix: [90%], [50%]
4. Proportion of error variance in $\textbf{X}$ (PEV): [0%], [10%], [50%]

The following algorithm provides how the initial data matrix is generated with correlated variables. Aside from the generation of the initial matrix, the data generating schemes are the same as the three schemes used in Section 3. Hence, after the generation of the initial matrix, the same steps given in Algorithms 1 and 2 are followed to simulate the data.

Fully crossing the factors in the study design led to $3 \times 2 \times 2 \times 3 = 36$ conditions, and 50 datasets were generated through the above schemes according to each condition. For each of the 1800 datasets, the 4 analysis methods were administered in the same manner as the above simulation study (Figs. 4, 5 and 6).

### Results

#### Proportion of variance accounted for (VAF)

Compared to Figs. 1, 2 and 3 from the simulation study in Section 3, it can be seen that throughout the three evaluation criteria that the new scheme with correlated initial data matrix leads to very similar results. The conclusions drawn are identical; the sparse weights model offers a much more complicated challenge for the sparse PCA methods in recovering the underlying true weights. Across all DGMs, SPCA methods underperform compared to USLPCA methods in identifying the zero-nonzero structure in the parameters.

## Appendix C: Autism gene expression data analysis with 36 non-zero coefficients per component

Since three components are extracted by sparse PCA from a dataset with 107 important probes out of the total 1107 probes, it may also be sensible to estimate the 36 non-zero coefficients per component. This would evenly distribute the 107 important probes across the three components. As done above, the results obtained from the different sparse PCA methods were compared against each other. Table 4 presents the proportion of non-zero coefficients that correspond across each pair of the four methods (above) and the Tucker congruence values between the component scores computed by the methods (below).

**Table 4:** Autism dataset

	SPCA-svd	SPCA-multi	USLPCA-svd
Proportion of corresponding non-zero coefficients			
SPCA-multi	0.231		
USLPCA-svd	0.204	0.185	
USLPCA-multi	0.074	0.056	0.417
Component scores Tucker congruence			
SPCA-multi	0.772		
USLPCA-svd	0.816	0.736	
USLPCA-multi	0.613	0.625	0.817

Above: proportion of corresponding non-zero coefficients out of the total 108 (36 non-zero coefficients $\times 3$ components). Below: Tucker congruence between the component scores

Table 4 conveys a message very much in line with the results obtained from 107 non-zero coefficients per component (Table 3). The four models construct different models. Only 23.1% of the non-zero weights found by SPCA-svd and SPCA-multi corresponded to each other. Likewise, 18.5% of the non-zero loadings obtained from USLPCA-svd were also obtained from USLPCA-multi. The proportion of corresponding non-zero coeffcients are also low when considering other pairs of methods. Component scores’ congruence values were all lower than 0.85 which is also in line with Table 3. The components extracted by the four methods would be interpreted as being different from each other.

With respect to the proportion of explained variance, SPCA-svd and SPCA-multi both recovered 36.6% of the data. Estimating a smaller number of non-zero weights did not decrease the amount of explained variance, compared to the model found above with 107 non-zero weights per component. This is in line with previous findings in the literature that showed that high levels of sparsity in weights can still explain large amount of variance (de Schipper & Van Deun, 2021). In contrast, USLPCA-svd and USLPCA-multi resulted in 6.5% and 7.4%. As in the simulation study and also for the results above, the sparse loadings methods expalin a considerably lower amount of variance than the sparse weights methods.

## Footnotes

1 The component weights function as the regression weights in the regression of the *r*th component score vector on the *J* observed variables. Adachi and Trendafilov (2016) also refers to the $\textbf{W}_R$ matrix as the weights matrix following the same rationale.

2 This is scaling both the variables and the component scores to unit variance which is common practice in the social and behavioral sciences and implemented in the PCA procedure of the SPSS software IBM Corp. (2013).

3 The entire solution path for elastic net can be generated when solved by the LARS algorithm and thus when the desired number of non-zero predictors are included in the model, the iteration procedure can be stopped.

4 A similar version with sparseness of the loadings obtained by adding a penalty has also been proposed (sPCA-rSVD; Shen and Huang (2008)). In this approach the orthogonality of the components is not imposed as the approach uses deflation to extract more than one component.

5 For the same reason of generating the data without noise, two zero weights were estimated for the first component.

6 The eigenvectors of the covariance matrix $\mathbf {\Sigma } = I^{-1}\textbf{X}^{\top } \textbf{X}$ are equal to the right singular vectors of $\textbf{X}$.

7 Referred to as ‘factor model’ in Fan et al. (2013)

8 For example, consider a case where $\textbf{X}$ consists of two variables which are identical to each other in a one-component setting. For the component scores, as long as the weights sum up to a particular value, there are infinitely many possible values that these weights can take; the linear combination $\textbf{X} \textbf{w}$ would always be identical.

9 The data is publicly available on the NCBI GEO database with the accession code GSE7329.

10 As it may also be sensible to distribute the non-zero coefficients evenly across the 3 components, we also administered the methods with 36 non-zero coefficients per component. The results can be found in Appendix C and the conclusions drawn are in line with the results presented here.

## Code Availability

The implementation of SCD-Cov-logR was done in R and Rcpp, which can be found on Github: https://github.com/soogs/Sparse-PCA-Critical-Assessment.
